# The contribution of vitamin D insufficiency to the onset of steatotic liver disease among individuals with metabolic dysfunction

**DOI:** 10.1038/s41598-024-57380-9

**Published:** 2024-03-20

**Authors:** Su-bin Lee, Mi Hyeon Jin, Jeong-Hyun Yoon

**Affiliations:** 1 Department of Pharmacy, Samsung Changwon Hospital, Sungkyunkwan University School of Medicine, Changwon, South Korea; 2 Department of Research Support, Samsung Changwon Hospital, School of Medicine, Sungkyunkwan University, Changwon, Korea; 3 College of Pharmacy and Research Institute for Drug Development, Pusan National University, 2, Busandaehak-ro, 63 Beon-gil, Geumjeong-gu, Busan, 46241 South Korea

**Keywords:** Hepatic steatosis, Metabolic syndrome, Metabolic associated fatty liver disease, Vitamin D insufficiency, Calcium and vitamin D, Non-alcoholic fatty liver disease

## Abstract

The interplay between fatty liver disease (FLD) and metabolic dysfunction has given rise to the concept of metabolic associated fatty liver disease (MAFLD). With vitamin D insufficiency frequently co-occurring with FLD and linked to metabolic abnormalities, this study investigates the potential role of vitamin D in the development of MAFLD. In this cross-sectional analysis, 22,476 participants with baseline metabolic dysfunction and known serum 25-OH-vitamin D3 levels were examined. The fatty liver index (FLI) was utilized to predict FLD, dividing subjects into MAFLD and non-MAFLD groups. Further stratification by vitamin D levels (sufficient vs. insufficient) and gender provided a detailed assessment through binary logistic regression to determine the association of vitamin D status with MAFLD incidence. Vitamin D insufficiency correlated with a higher MAFLD incidence in metabolically impaired individuals. Post-adjustment, the correlation was stronger (men: aOR = 1.32, 95% CI = 1.22–1.43, *P* < 0.001; women: aOR = 1.53, 95% CI = 1.18–1.98, *P* = 0.001). Lower serum 25-OH-vitamin D3 levels were found in MAFLD patients across genders (men: *P* = 0.003; women: *P* = 0.014), with a higher prevalence of insufficiency in MAFLD cases (men: *P* = 0.007; women: *P* = 0.003). The vitamin D-MAFLD link was stable across subgroups and using varying FLI criteria. Our findings indicate a clear association between vitamin D insufficiency and increased MAFLD incidence, underscoring the potential of vitamin D as an anti-lipogenic and anti-fibrotic agent.

## Introduction

The prevalence of nonalcoholic fatty liver disease (NAFLD) has surged, positioning it as a principal contributor to liver pathology globally and a significant factor in hepatocellular carcinoma within the United States^1^. Concurrent with the rise in type 2 diabetes mellitus (T2DM), obesity, and metabolic syndrome (MetS), NAFLD's prevalence has also escalated, earning the designation as a hepatic component of MetS. The mortality risk associated with NAFLD is notably amplified by the co-occurrence of metabolic derangements, underlining the critical role of metabolic factors in its pathogenesis^2^. Given the intricate connection between metabolic dysfunction and the onset of fatty liver diseases, there has been a call for a revised diagnostic paradigm. The existing definition of NAFLD is deemed insufficient as it fails to incorporate a set of "positive" criteria that capture the essential metabolic features^3^. Consequently, a new term, metabolic associated fatty liver disease (MAFLD), has been proposed by a consortium of international experts, signifying a shift from the traditional NAFLD nomenclature to better reflect the current scientific understanding^4^.

The portrayal of NAFLD merely as a manifestation of MetS is subject to ongoing debate. Epidemiological findings reveal that MetS does not invariably lead to NAFLD, and the linkage between hepatic steatosis and atherosclerotic cardiovascular disease (ASCVD) persists even after controlling for MetS risk factors^5–7^. This observation alludes to the potential of fatty liver as a distinct marker for ASCVD independent of other metabolic dysfunctions. The role of vitamin D extends beyond its well-established influence on bone metabolism, exerting broad effects on multiple organ systems^8^. Recently, vitamin D's protective potential against NAFLD development and its progression has attracted significant research interest, fueled by indications that vitamin D may modulate the various phases of NAFLD progression, including its regulatory impact on metabolism, and anti-steatotic, anti-fibrotic, and anti-inflammatory effects^8,9^. Observations from earlier studies suggest that vitamin D deficiency could play a role in the etiology of NAFLD^10,11^. However, the correlation between vitamin D status and fatty liver disease in the context of metabolic dysfunction is not well-established. This study aims to delineate the relationship between serum vitamin D levels and the incidence of MAFLD.

## Results

### Baseline characteristics by gender

Table 1 displays the demographic and clinical baseline characteristics of the participants. The cohort predominantly consisted of men (72.61%), reflecting the occupational demographics of the region. Notable gender disparities were observed in MAFLD incidence (53.27% in men vs. 12.96% in women, *P* < 0.001) and baseline characteristics. Given alcohol's established association with liver disease and the observed gender differences in alcohol consumption, the analysis was conducted separately for 16,320 men and 6156 women.

**Table 1:** Baseline characteristics of the study population by gender.

	Overall (n = 22,476)	Men (n = 16,320)	Women (n = 6156)	*P* value
Sex (n, %)				< 0.001
Men	16,320 (72.61)			
Women	6156 (27.39)			
Age, years (mean ± SD)	39.52 ± 11.26	38.77 ± 10.53	41.49 ± 12.79	< 0.001
Smoking status (n, %)				0.001
Never	9,069 (44.72)	4,394 (28.93)	4,675 (91.90)	
Former	4,150 (20.47)	3,943 (25.96)	207 (4.07)	
Current	7,059 (34.81)	6,854 (45.12)	205 (4.03)	
Alcohol consumption (n, %)				< 0.001
Never	647 (3.47)	396 (2.69)	251 (6.39)	
Moderate	13,783 (73.92)	10,478 (71.19)	3,305 (84.16)	
Heavy	4,216 (22.61)	3,845 (26.12)	371 (9.45)	
Physical activity (n, %)				< 0.001
Inactive	18,151 (83.71)	13,037 (82.75)	5,114 (86.24)	
Moderately active	1,830 (8.44)	1,379 (8.75)	451 (7.61)	
HEPA active	1,703 (7.85)	1,338 (8.49)	365 (6.16)	
Abdominal obesity (mean ± SD)				
BMI (kg/m^2^)	26.07 ± 3.60	26.54 ± 3.50	24.82 ± 3.58	< 0.001
WC (cm)	88.68 ± 9.70	90.20 ± 9.52	84.64 ± 8.97	< 0.001
Blood pressure (mean ± SD)				
SBP (mmHg)	126.98 ± 15.78	128.31 ± 15.31	123.44 ± 16.47	< 0.001
DBP (mmHg)	77.30 ± 10.83	78.08 ± 10.57	75.23 ± 11.22	< 0.001
Hypertension (n, %)	6,958 (30.96)	5,459 (33.45)	1,499 (24.35)	< 0.001
Glucose intolerance index (mean ± SD)				
FPG (mg/dL)	97.40 ± 21.25	98.30 ± 21.52	95.03 ± 20.32	< 0.001
HbA1C (%)	5.68 ± 0.78	5.70 ± 0.79	5.66 ± 0.75	< 0.001
HOMA-IR score	1.83 ± 1.55	1.85 ± 1.56	1.79 ± 1.51	0.023
Diabetes (n, %)	2,014 (8.96)	1,546 (9.47)	468 (7.60)	< 0.001
Lipid profile (mean ± SD)				
TC (mg/dL)	196.69 ± 38.52	198.64 ± 38.80	191.53 ± 37.28	< 0.001
TG (mg/dL)	153.65 ± 134.26	171.33 ± 147.72	106.75 ± 70.26	< 0.001
HDL-C (mg/dL)	53.65 ± 13.84	51.31 ± 12.68	59.85 ± 14.82	< 0.001
LDL-C (mg/dL)	126.79 ± 35.28	128.39 ± 35.32	122.58 ± 34.83	< 0.001
AST, IU/L (mean ± SD)	26.69 ± 22.15	28.67 ± 24.03	21.45 ± 14.94	< 0.001
ALT, IU/L (mean ± SD)	32.09 ± 31.13	36.68 ± 33.20	19.92 ± 20.28	< 0.001
GGT, IU/L (mean ± SD)	42.87 ± 55.32	51.49 ± 60.73	20.04 ± 25.99	< 0.001
MAFLD (n, %)	9,492 (42.23)	8,694 (53.27)	798 (12.96)	< 0.001
Viral hepatitis (n, %)	770 (3.44)	582 (3.59)	188 (3.05)	0.048
Vitamin D insufficiency (n, %)	15,117 (67.26)	10,620 (65.07)	4,497 (73.05)	< 0.001
Vitamin D, ng/mL (mean ± SD)	17.81 ± 7.92	18.23 ± 7.39	16.67 ± 9.06	< 0.001

Values are presented as mean ± standard deviation or number (%) by descriptive analysis, frequency analysis. *P* values were calculated using independent t-test or chi-square test. The number of n of each variable may differ if study values are missing. Abbreviations: ALT, alanine transaminase; AST, aspartate transaminase; BMI, body mass index; DBP, diastolic blood pressure; FPG, fasting plasma glucose; GGT, gamma glutamyl transferase; HbA1c, hemoglobin A1c; HDL-C, high-density lipoprotein cholesterol; HEPA, health-enhancing physical activity; HOMA-IR, homeostasis model assessment-insulin resistance; LDL-C, low-density lipoprotein cholesterol; MAFLD, metabolic associated fatty liver disease; SBP, systolic blood pressure; TC, total cholesterol; TG, triglyceride; WC, waist circumference.

Men were significantly younger than women (38.77 ± 10.53 years in men vs. 41.49 ± 12.79 years in women, *P* < 0.001). The prevalence of current smoking was substantially higher in men (45.12% vs. 4.03% in women, *P* = 0.001). The proportion of heavy drinkers was also significantly higher in men (26.12% vs. 9.45% in women, *P* < 0.001), as was the engagement in physically active lifestyles (8.46% in men vs. 6.16% in women, *P* < 0.001). Men had significantly higher rates of hypertension (33.45% vs. 24.35% in women, *P* < 0.001) and diabetes (9.47% vs. 7.60% in women, *P* < 0.001). Additionally, men presented with higher levels of total cholesterol (TC), triglycerides (TG), low-density lipoprotein cholesterol (LDL-C), aspartate aminotransferase (AST), alanine aminotransferase (ALT), gamma-glutamyl transferase (GGT), body mass index (BMI), waist circumference (WC), fasting plasma glucose (FPG), hemoglobin A1c (HbA1c), and homeostatic model assessment for insulin resistance (HOMA-IR) scores, alongside lower high-density lipoprotein cholesterol (HDL-C) levels. Women had significantly lower serum vitamin D levels (18.23 ± 7.39 ng/mL in men vs. 16.67 ± 9.06 ng/mL in women, *P* < 0.001), correlating with a higher prevalence of vitamin D insufficiency (65.07% in men vs. 73.05% in women, *P* < 0.001).

### Baseline characteristics based on vitamin D status

Table 2 details the baseline characteristics of participants stratified by vitamin D status. Among the 16,320 men and 6156 women who met the inclusion criteria, 5700 men and 1659 women were categorized into the vitamin D sufficiency group, while 10,620 men and 4497 women comprised the insufficiency group.

**Table 2:** Baseline characteristics by vitamin D status in men and women of the study population.

	Men (n = 16,320)			Women (n = 6,156)		
	Vitamin D		*P* value	Vitamin D		*P* value
	Sufficiency (n = 5,700)	Insufficiency (n = 10,620)		Sufficiency (n = 1,659)	Insufficiency (n = 4,497)	
Age, years (mean ± SD)	41.98 ± 11.33	37.05 ± 9.64	< 0.001	48.77 ± 13.15	38.81 ± 11.55	< 0.001
Smoking status (n, %)			< 0.001			< 0.001
Never	1,332 (25.36)	3,062 (30.81)		1,323 (95.11)	3,352 (90.69)	
Former	1,607 (30.60)	2,336 (23.50)		35 (2.52)	172 (4.65)	
Current	2,313 (44.04)	4,541 (45.69)		33 (2.37)	172 (4.65)	
Alcohol consumption (n, %)			< 0.001			0.001
Never	114 (2.25)	282 (2.92)		363 (3.84)	215 (7.19)	
Moderate	3,429 (67.70)	7,049 (73.02)		819 (87.31)	2,486 (83.17)	
Heavy	1,522 (30.05)	2,323 (24.06)		83 (8.85)	288 (9.64)	
Physical activity (n, %)			< 0.001			0.019
Inactive	4,347 (79.17)	8,690 (84.67)		1,342 (84.56)	3,772 (86.85)	
Moderately active	551 (10.03)	828 (8.07)		146 (9.20)	305 (7.02)	
HEPA active	593 (10.80)	745 (7.26)		99 (6.24)	266 (6.12)	
Abdominal obesity (mean ± SD)						
BMI (kg/m^2^)	26.32 ± 3.34	26.66 ± 3.58	< 0.001	24.13 ± 3.21	25.08 ± 3.67	< 0.001
WC (cm)	89.70 ± 9.24	90.47 ± 9.66	< 0.001	83.73 ± 8.06	84.98 ± 9.26	< 0.001
Blood pressure (mean ± SD)						
SBP (mmHg)	128.96 ± 15.99	127.97 ± 14.92	< 0.001	125.89 ± 17.64	122.54 ± 15.92	< 0.001
DBP (mmHg)	77.66 ± 10.81	78.30 ± 10.43	< 0.001	76.34 ± 11.65	74.82 ± 11.03	< 0.001
Hypertension, (n, %)	2,178 (38.21)	3,281 (30.89)	< 0.001	585 (35.26)	914 (20.32)	< 0.001
Glucose intolerance index (mean ± SD)						
FPG (mg/dL)	98.95 ± 20.639	97.95 ± 21.95	0.005	98.10 ± 19.94	93.90 ± 20.34	< 0.001
HbA1C (%)	5.72 ± 0.75	5.68 ± 0.80	0.011	5.76 ± 0.73	5.62 ± 0.76	< 0.001
HOMA-IR score	1.78 ± 1.42	1.90 ± 1.66	< 0.001	1.81 ± 1.49	1.78 ± 1.52	0.578
Diabetes (n, %)	635 (11.14)	911 (8.58)	< 0.001	186 (11.21)	282 (6.27)	< 0.001
Lipid profile (mean ± SD)						
TC (mg/dL)	198.33 ± 38.58	198.81 ± 38.92	0.454	196.30 ± 39.05	189.77 ± 36.45	< 0.001
TG (mg/dL)	161.44 ± 124.04	176.64 ± 158.74	< 0.001	106.72 ± 64.36	106.77 ± 72.33	0.981
HDL-C (mg/dL)	52.73 ± 13.18	50.55 ± 12.34	< 0.001	62.39 ± 15.93	58.91 ± 14.28	< 0.001
LDL-C (mg/dL)	129.19 ± 35.68	127.96 ± 35.13	0.034	126.69 ± 37.25	121.06 ± 33.77	< 0.001
AST, IU/L (mean ± SD)	29.38 ± 25.99	28.28 ± 22.90	0.005	23.28 ± 12.41	20.77 ± 15.72	< 0.001
ALT, IU/L (mean ± SD)	35.93 ± 34.69	37.07 ± 32.37	0.036	21.36 ± 17.55	19.39 ± 21.18	< 0.001
GGT, IU/L (mean ± SD)	55.18 ± 72.48	49.50 ± 53.28	< 0.001	22.27 ± 29.09	19.22 ± 24.69	< 0.001
MAFLD (n, %)	2,954 (51.82)	5,740 (54.05)	0.007	180 (10.85)	618 (13.74)	0.003
Viral hepatitis (n, %)	246 (4.34)	336 (3.19)	< 0.001	61 (3.68)	127 (2.82)	0.084
Vitamin D, ng/mL (mean ± SD)	26.23 ± 5.88	13.94 ± 3.56	< 0.001	28.47 ± 8.47	12.32 ± 3.96	< 0.001

Values are presented as mean ± standard deviation or number (%) by descriptive analysis, frequency analysis. *P* values were calculated using independent t-test or chi-square test. Abbreviations: ALT, alanine transaminase; AST, aspartate transaminase; BMI, body mass index; DBP, diastolic blood pressure; FPG, fasting plasma glucose; HbA1c, hemoglobin A1c; HEPA, health-enhancing physical activity; GGT, gamma glutamyl transferase; HDL-C, high-density lipoprotein cholesterol; HOMA-IR, homeostasis model assessment-insulin resistance; LDL-C, low-density lipoprotein cholesterol; MAFLD, metabolic associated fatty liver disease; SBP, systolic blood pressure; TC, total cholesterol; TG, triglyceride; WC, waist circumference.

In the vitamin D sufficiency group, men were significantly older compared to those in the insufficiency group (41.98 ± 11.33 vs. 37.05 ± 9.64 years; *P* < 0.001). The proportion of never-smokers was higher in the sufficiency group (25.36% vs. 30.81% in the insufficiency group; *P* < 0.001). Heavy drinking was more prevalent among sufficient men (30.05% vs. 24.06% in the insufficiency group; *P* < 0.001), as was engagement in physical activities (10.80% vs. 7.26%; *P* < 0.001). There were also significantly higher incidences of hypertension (38.21% vs. 30.89% in the insufficiency group; *P* < 0.001) and diabetes (11.14% vs. 8.58%; *P* < 0.001). Men in the sufficiency group had higher levels of LDL-C, HDL-C, AST, GGT, FPG, and HbA1c but lower BMI, WC, TG, ALT, and HOMA-IR scores. A higher incidence of MAFLD was observed in the insufficiency group compared to the sufficiency group (54.05% vs. 51.82%; *P* = 0.007), and viral hepatitis was more prevalent among the sufficient (4.34% vs. 3.19%; *P* < 0.001).

For women, those in the vitamin D sufficiency group were significantly older than their insufficient counterparts (48.77 ± 13.15 vs. 38.81 ± 11.55 years; *P* < 0.001) and had a higher proportion of never-smokers (95.11% vs. 90.69%; *P* < 0.001). Vitamin D insufficient women were more likely to abstain from alcohol (7.19% vs. 3.84%; *P* = 0.001) and were less physically active (86.85% vs. 84.56%; *P* = 0.019). Hypertension (35.26% vs. 20.32%; *P* < 0.001) and diabetes (11.21% vs. 6.27%; *P* < 0.001) were more frequent in the sufficiency group. Additionally, women with sufficient vitamin D levels had higher TC, HDL-C, LDL-C, AST, ALT, GGT, FPG, HbA1c levels but lower BMI and WC. The MAFLD incidence was greater in the insufficiency group (13.74% vs. 10.85%; *P* = 0.003). The average serum vitamin D levels were 26.23 ± 5.88 ng/mL for sufficient men and 13.94 ± 3.56 ng/mL for insufficient men; for women, these levels were 28.47 ± 8.47 ng/mL and 12.32 ± 3.96 ng/mL, respectively. Contrary to men, no significant difference in the prevalence of viral hepatitis was observed between the sufficiency and insufficiency groups among women.

### Comparison of characteristics in individuals with and without MAFLD

Characteristics of the study population stratified by the presence of MAFLD are presented in Table 3. Among men, MAFLD was significantly more common in younger individuals, smokers, drinkers, and those less physically active. They exhibited significantly higher BMI, WC, SBP, DBP, FPG, HbA1c, and levels of all lipid profiles except HDL-C, as well as AST, ALT, and GGT. The MAFLD group had a lower mean serum vitamin D level (18.07 ± 7.00 ng/mL) compared to the non-MAFLD group (18.41 ± 7.81 ng/mL, *P* = 0.003), which coincided with a higher vitamin D insufficiency rate (66.02% vs. 63.99%, *P* = 0.007). In women, the mean serum vitamin D level was also significantly lower in the MAFLD group (15.93 ± 8.02 ng/mL) than in the non-MAFLD group (16.78 ± 9.21 ng/mL, *P* = 0.014), and a higher rate of vitamin D insufficiency was observed (77.44% vs. 72.40%, *P* = 0.003).

**Table 3:** MAFLD prevalence and associated characteristics in men and women of the study population.

	Men (n = 16,320)			Women (n = 6,156)		
	MAFLD		*P* value	MAFLD		*P* value
	Yes (n = 8,694)	No (n = 7,626)		Yes (n = 798)	No (n = 5,358)	
Age, years (mean ± SD)	38.39 ± 8.75	39.21 ± 12.23	< 0.001	40.74 ± 11.87	41.60 ± 12.92	0.077
Smoking status (n, %)			< 0.001			0.001
Never	1,891 (23.22)	2,503 (35.51)		602 (88.79)	4,073 (92.38)	
Former	2,015 (24.75)	1,928 (27.36)		32 (4.72)	175 (3.97)	
Current	4,237 (52.03)	2,617 (37.13)		44 (6.49)	161 (3.65)	
Alcohol consumption (n, %)			< 0.001			0.002
Never	140 (1.75)	256 (3.81)		25 (4.63)	226 (6.67)	
Moderate	5,187 (64.77)	5,300 (78.81)		444 (82.22)	2,861 (84.47)	
Heavy	2,676 (33.48)	1,169 (17.38)		71 (13.15)	300 (8.86)	
Physical activity (n, %)			< 0.001			0.695
Inactive	7,038 (83.95)	5,999 (81.40)		673 (87.18)	4,441 (86.10)	
Moderately active	766 (9.14)	613 (8.32)		56 (7.25)	395 (7.66)	
HEPA active	580 (6.92)	758 (10.28)		43 (5.57)	322 (6.24)	
Abdominal obesity (mean ± SD)						
BMI (kg/m^2^)	28.65 ± 3.01	24.13 ± 2.24	< 0.001	31.21 ± 3.67	23.87 ± 2.39	< 0.001
WC (cm)	96.35 ± 7.80	83.20 ± 5.71	< 0.001	100.90 ± 8.70	82.22 ± 5.99	< 0.001
Blood pressure (mean ± SD)						
SBP (mmHg)	130.83 ± 15.29	125.44 ± 14.81	< 0.001	131.77 ± 16.20	122.20 ± 16.14	< 0.001
DBP (mmHg)	79.98 ± 10.63	75.90 ± 10.06	< 0.001	80.78 ± 11.15	74.40 ± 10.99	< 0.001
Glucose intolerance index (mean ± SD)						
FPG (mg/dL)	100.22 ± 23.22	96.11 ± 19.17	< 0.001	106.43 ± 34.24	93.33 ± 16.66	< 0.001
HbA1C (%)	5.76 ± 0.81	5.63 ± 0.75	< 0.001	6.06 ± 1.19	5.60 ± 0.64	< 0.001
Lipid profile (mean ± SD)						
TC (mg/dL)	211.03 ± 38.02	184.51 ± 34.64	< 0.001	205.92 ± 39.37	189.39 ± 36.48	< 0.001
TG (mg/dL)	239.13 ± 170.19	94.05 ± 49.67	< 0.001	187.12 ± 122.21	94.78 ± 48.43	< 0.001
HDL-C (mg/dL)	47.24 ± 10.50	55.95 ± 13.35	< 0.001	52.09 ± 12.71	61.00 ± 14.77	< 0.001
LDL-C (mg/dL)	136.10 ± 35.83	119.58 ± 32.58	< 0.001	134.20 ± 36.49	120.85 ± 34.24	< 0.001
AST, IU/L (mean ± SD)	34.64 ± 26.60	23.00 ± 19.20	< 0.001	34.24 ± 28.92	19.69 ± 10.40	< 0.001
ALT, IU/L (mean ± SD)	48.92 ± 39.53	22.72 ± 14.54	< 0.001	40.40 ± 41.29	16.86 ± 12.10	< 0.001
GGT, IU/L (mean ± SD)	76.63 ± 73.52	22.82 ± 13.75	< 0.001	50.48 ± 59.59	15.51 ± 9.44	< 0.001
Vitamin D insufficiency (n, %)	5,740 (66.02)	4,880 (63.99)	0.007	618 (77.44)	3,879 (72.40)	0.003
Vitamin D, ng/mL (mean ± SD)	18.07 ± 7.00	18.41 ± 7.81	0.003	15.93 ± 8.02	16.78 ± 9.21	0.014

Values are presented as mean ± standard deviation or number (%) by descriptive analysis, frequency analysis. *P* values were calculated using independent t-test or chi-square test. Abbreviations: ALT, alanine transaminase; AST, aspartate transaminase; BMI, body mass index; DBP, diastolic blood pressure; FPG, fasting plasma glucose; GGT, gamma-glutamyl transferase; HbA1c, hemoglobin A1c; HDL-C, high-density lipoprotein cholesterol; HEPA, health-enhancing physical activity; LDL-C, low-density lipoprotein cholesterol; SBP, systolic blood pressure; TC, total cholesterol; TG, triglyceride; WC, waist circumference.

### Comparison of characteristics according to vitamin D status in MAFLD group

Table 4 compares the characteristics of MAFLD subjects based on vitamin D status. Men with sufficient vitamin D had mean serum levels of 25.85 ± 5.31 ng/mL, while those with insufficiency had levels of 14.07 ± 3.55 ng/mL. The sufficiency group was significantly older and had higher rates of smoking, drinking, physical activity, hypertension, and diabetes. This group also had lower BMI, WC, TC, and TG levels, but higher HDL-C, AST, and GGT levels. Women with sufficient vitamin D levels (27.48 ± 7.67 ng/mL) compared to those with insufficiency (12.57 ± 3.98 ng/mL) were older, more likely to be non-smokers, and had a higher prevalence of hypertension. Unlike men, the sufficiency group in women did not have significant differences in alcohol consumption and physical activity when compared to the insufficiency group, though the smoking rate was significantly lower.

**Table 4:** Comparison of characteristics according to vitamin D status in Men and Women with MAFLD.

	Men (n = 8,694)			Women (n = 798)		
	Vitamin D		*P* value	Vitamin D		*P* value
	Sufficiency (n = 2,954)	Insufficiency (n = 5,740)		Sufficiency (n = 180)	Insufficiency (n = 618)	
Age, years (mean ± SD)	41.01 ± 9.22	37.04 ± 8.18	< 0.001	46.38 ± 12.19	39.10 ± 11.27	< 0.001
Smoking status (n, %)			< 0.001			0.013
Never	513 (18.70)	1,378 (25.52)		141 (95.27)	461 (86.98)	
Former	769 (28.03)	1,246 (23.07)		2 (1.35)	30 (5.66)	
Current	1,461 (53.26)	2,776 (51.41)		5 (3.38)	39 (7.36)	
Alcohol consumption (n, %)			< 0.001			0.115
Never	35 (1.30)	105 (1.98)		3 (2.70)	22 (5.13)	
Moderate	1,623 (60.29)	3,555 (67.05)		99 (89.19)	345 (80.42)	
Heavy	1,034 (38.41)	1,642 (30.97)		9 (8.11)	62 (14.45)	
Physical activity (n, %)			< 0.001			0.263
Inactive	2,312 (81.35)	4,726 (85.28)		148 (87.06)	525 (87.21)	
Moderately active	284 (9.99)	482 (8.70)		9 (5.29)	47 (7.81)	
HEPA active	246 (8.66)	334 (6.03)		13 (7.65)	30 (4.98)	
Abdominal obesity (mean ± SD)						
BMI (kg/m^2^)	28.38 ± 2.87	28.79 ± 3.07	< 0.001	30.08 ± 3.49	31.54 ± 3.66	< 0.001
WC (cm)	95.82 ± 7.67	96.62 ± 7.85	< 0.001	98.79 ± 8.57	101.52 ± 8.64	< 0.001
Blood pressure (mean ± SD)						
SBP (mmHg)	131.40 ± 15.78	130.54 ± 15.03	0.014	133.61 ± 15.84	131.24 ± 16.28	0.085
DBP (mmHg)	79.65 ± 10.74	80.16 ± 10.58	0.035	81.44 ± 11.26	80.59 ± 11.13	0.368
Hypertension, (n, %)	1,255 (42.48)	2,145 (37.37)	< 0.001	95 (52.78)	230 (37.22)	< 0.001
Glucose intolerance index (mean ± SD)						
FPG (mg/dL)	100.71 ± 22.70	99.96 ± 23.48	0.156	108.85 ± 32.67	105.73 ± 34.67	0.282
HbA1C (%)	5.76 ± 0.78	5.75 ± 0.83	0.607	6.07 ± 1.04	6.06 ± 1.24	0.909
HOMA-IR score	2.33 ± 1.60	2.43 ± 1.69	0.024	3.66 ± 2.71	3.60 ± 2.67	0.798
Diabetes (n, %)	321 (10.87)	533 (9.29)	0.019	41 (22.78)	110 (17.80)	0.133
Lipid profile (mean ± SD)						
TC (mg/dL)	209.73 ± 37.65	211.70 ± 38.19	0.022	207.07 ± 45.02	205.58 ± 37.59	0.656
TG (mg/dL)	226.28 ± 137.60	245.74 ± 184.40	< 0.001	181.27 ± 110.23	188.83 ± 125.51	0.465
HDL-C (mg/dL)	48.37 ± 10.95	46.65 ± 10.21	< 0.001	55.26 ± 14.22	51.17 ± 12.10	< 0.001
LDL-C (mg/dL)	136.44 ± 32.92	135.92 ± 35.78	0.525	134.88 ± 41.65	134.00 ± 34.88	0.774
AST, IU/L (mean ± SD)	34.59 ± 33.60	33.14 ± 22.13	0.016	33.11 ± 20.26	33.28 ± 31.01	0.946
ALT, IU/L (mean ± SD)	47.82 ± 43.20	49.48 ± 37.50	0.064	40.27 ± 36.71	40.44 ± 42.55	0.961
GGT, IU/L (mean ± SD)	83.87 ± 90.45	72.90 ± 62.75	< 0.001	61.81 ± 72.09	47.18 ± 55.05	0.004
Viral hepatitis (n, %)	94 (3.19)	161 (2.83)	0.343	7 (3.89)	14 (2.27)	0.231
Vitamin D, ng/mL (mean ± SD)	25.85 ± 5.31	14.07 ± 3.55	< 0.001	27.48 ± 7.67	12.57 ± 3.98	< 0.001

Values are presented as mean ± standard deviation or number (%) by descriptive analysis, frequency analysis. *P* values were calculated using independent t-test or chi-square test. Abbreviations: ALT, alanine transaminase; AST, aspartate transaminase; BMI, body mass index; DBP, diastolic blood pressure; FPG, fasting plasma glucose; GGT, gamma-glutamyl transferase; HbA1c, hemoglobin A1c; HDL-C, high-density lipoprotein cholesterol; HEPA, health-enhancing physical activity; HOMA-IR, homeostasis model assessment-insulin resistance; LDL-C, low-density lipoprotein cholesterol; SBP, systolic blood pressure; TC, total cholesterol; TG, triglyceride; WC, waist circumference.

### Association between MAFLD and vitamin D status in subjects with metabolic dysfunction

Binary logistic regression was conducted to assess the relationship between vitamin D status and MAFLD incidence, with findings detailed in Table 5. The analysis revealed a significant association between vitamin D insufficiency and MAFLD incidence, with men showing an odds ratio (OR) of 1.09 (95% CI: 1.03–1.17, *P* = 0.007) and women an OR of 1.31 (95% CI: 1.10–1.56, *P* = 0.003). After adjusting for potential confounders, the association persisted; in men, the adjusted OR (aOR) ranged from 1.06 (95% CI: 0.99–1.13, *P* = 0.099) to 1.31 (95% CI: 1.22–1.41, *P* < 0.001), and in women from 1.28 (95% CI: 1.06–1.54, *P* = 0.010) to 1.53 (95% CI: 1.20–1.96, *P* = 0.001) across various models. The strongest associations were noted in the fully adjusted model (Model 4), which included age, sex, time of health checkup, alcohol intake, physical activity, and smoking status (men: aOR = 1.32, 95% CI: 1.22–1.43, *P* < 0.001; women: aOR = 1.53, 95% CI: 1.18–1.98, *P* = 0.001). Building on our initial findings, we further refined our analysis to include BMI adjustments. The newly included Model 5, stratified by a BMI threshold of 23, maintains the significant association between vitamin D insufficiency and the incidence of MAFLD. Specifically, this BMI-adjusted model revealed that men with vitamin D insufficiency had an odds ratio (OR) of 1.43 (95% CI: 1.31–1.56, *P* < 0.001), and women had an OR of 1.34 (95% CI: 1.02–1.76, *P* = 0.034). These results reaffirm the strength of the association across various BMI categories.

**Table 5:** Odds ratios of MAFLD incidence according to vitamin D status.

	Crude		Model 1		Model 2		Model 3		Model 4		Model 5	
	OR (95% CI)	*P* value	OR (95% CI)	*P* value	OR (95% CI)	*P* value	OR (95% CI)	*P* value	OR (95% CI)	*P* value	OR (95% CI)	*P* value
Men												
Sufficiency	Reference		Reference		Reference		Reference		Reference		Reference	
Insufficiency	1.09 (1.03–1.17)	0.007	1.06 (0.99–1.13)	0.099	1.20 (1.12–1.29)	< 0.001	1.31 (1.22–1.41)	< 0.001	1.32 (1.22–1.43)	< 0.001	1.43 (1.31–1.56)	< 0.001
Women												
Sufficiency	Reference		Reference		Reference		Reference		Reference		Reference	
Insufficiency	1.31 (1.10–1.56)	0.003	1.28 (1.06–1.54)	0.010	1.60 (1.31–1.95)	< 0.001	1.53 (1.20–1.96)	0.001	1.53 (1.18–1.98)	0.001	1.34 (1.02–1.76)	0.034

Odds ratios and *P* values were calculated using binary logistic regression.

Model 1: Adjusted for age.

Model 2: Adjusted for Model 1 plus year of visit and quarter of season (spring, summer, fall, and winter).

Model 3: Adjusted for Model 2 plus alcohol intake (never, moderate, and heavy drinkers).

Model 4: Adjusted for Model 3 plus smoking status (never, former, and current) and physical activity (inactive, moderately active, and HEPA active).

Model 5: Adjusted for Model 4 plus BMI stratification (threshold of 23 kg/m²). Abbreviation: MAFLD, metabolic associated fatty liver disease; OR, odds ratio; CI, confidence interval; HEPA, health-enhancing physical activity; BMI, body mass index.

### Subgroup analyses stratifying by clinically relevant factors

**Figure 1:**
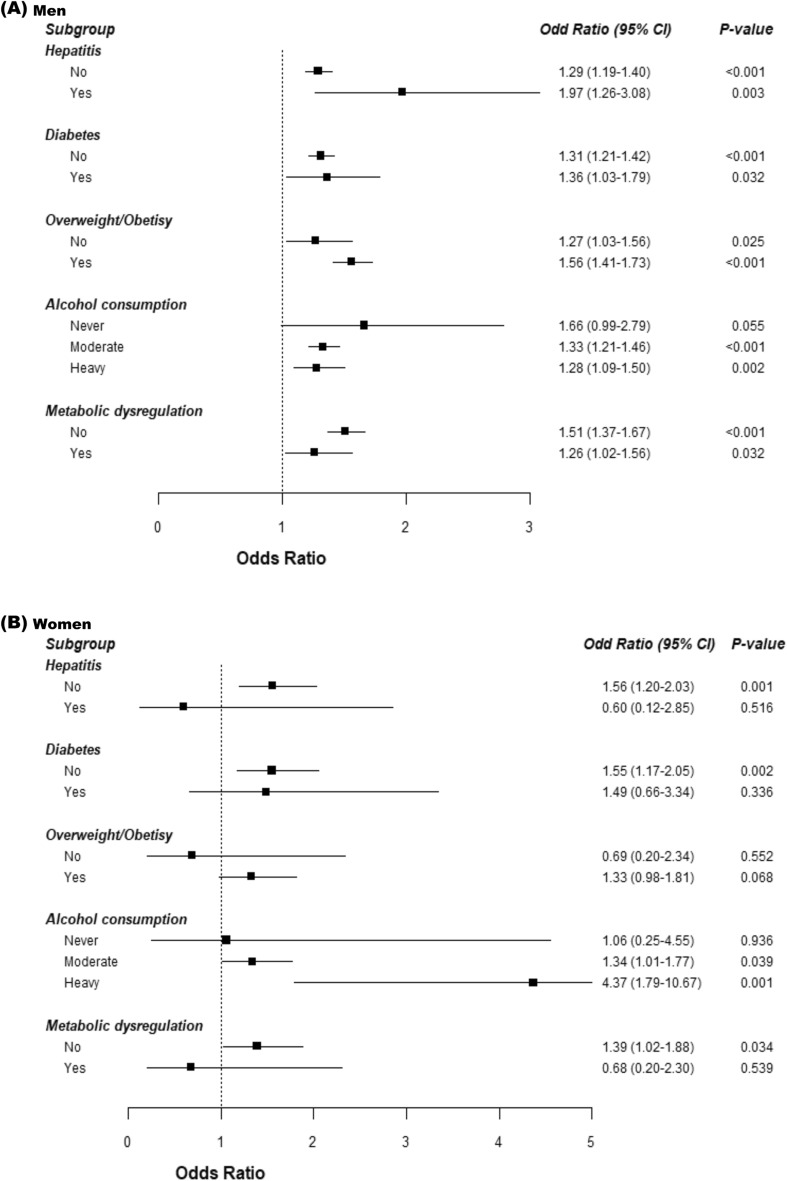
Incidence of MAFLD according to vitamin D status, (**A**) for men and (**B**) for women in clinically relevant subgroups.

Subgroup analyses were performed to examine the consistency of the association between vitamin D insufficiency and MAFLD across different clinical contexts, adjusting for all confounders in Model 4 as shown in Fig. 1. In men, vitamin D insufficiency was consistently associated with increased MAFLD incidence, irrespective of hepatitis, diabetes, being overweight/obese, or metabolic dysregulation in normal-weight individuals. Notably, in the context of hepatitis, vitamin D insufficiency significantly raised the incidence of MAFLD (OR = 1.97, 95% CI: 1.26–3.08, *P* = 0.003). Alcohol consumption also influenced the association, with vitamin D insufficiency correlating with a higher incidence of MAFLD in those who consume alcohol. Conversely, in women, the relationship between vitamin D insufficiency and MAFLD was less pronounced in subgroups with chronic hepatitis, diabetes, or metabolic dysregulation in normal-weight subjects. No association was found in women with or without overweight/obesity. However, vitamin D insufficiency was linked to a higher incidence of MAFLD in alcohol consumers, similar to men, with a pronounced association in heavy drinkers (OR = 4.37, 95% CI: 1.79–10.67, *P* = 0.001).

### Sensitivity analysis

A sensitivity analysis was conducted to evaluate the robustness of the association between vitamin D insufficiency and MAFLD. This study initially defined fatty liver based on a Fatty Liver Index (FLI) of 60 or higher, excluding individuals with an FLI between 30 to < 60. The sensitivity analysis utilized an alternative FLI threshold of ≥ 30 for a fatty liver diagnosis, as commonly adopted in Korean studies^12,13^. After classifying fatty liver with this lower cut-off and adjusting for all confounders outlined in Model 4 (details provided in Supplementary Table 1), the association of vitamin D insufficiency with MAFLD remained significant. In men, the adjusted odds ratio (aOR) was 1.25 (95% CI: 1.17–1.34, *P* < 0.001), and in women, it was 1.39 (95% CI: 1.18–1.64, *P* < 0.001), confirming that vitamin D insufficiency is a significant predictor of MAFLD regardless of the FLI threshold used.

## Discussion

In our population-based study, we explored the relationship between serum vitamin D levels and MAFLD incidence. The findings indicate that vitamin D insufficiency is associated with increased likelihood of MAFLD in individuals with metabolic dysfunction. Our data suggest that metabolic dysfunction may predispose subjects to MAFLD when accompanied by insufficient vitamin D levels. This study also found that subjects with MAFLD had notably lower mean serum vitamin D levels than those without the disease, leading to a significantly higher prevalence of vitamin D insufficiency in both men and women with MAFLD.

Vitamin D, primarily synthesized in the liver, acts as an important secosteroid hormone with pleiotropic effects^14^. Beyond its role in calcium and bone homeostasis, vitamin D is implicated in the regulation of cell proliferation and differentiation^15^, with relevance to the pathogenesis of chronic liver disease^16^. Evidence increasingly suggests a link, potentially causative, between vitamin D deficiency and NAFLD^17–19^. Consequently, vitamin D's anti-lipogenic, antifibrotic, and anti-inflammatory properties have been highlighted^20^. However, the debate persists over the significance of vitamin D status in NAFLD progression. Targher et al.'s study, the first to examine this relationship through biopsy-proven NAFLD, found lower vitamin D levels in NAFLD subjects compared to controls and an association between vitamin D status and NAFLD severity, with NASH patients exhibiting lower levels than those with isolated fatty liver^21^. Pacifico et al. conducted a systematic review of 45 studies, finding an inverse relationship between vitamin D status and NAFLD in over half the studies^21^. These findings align with our observations, which also demonstrated common vitamin D insufficiency in MAFLD subjects. Contrarily, Jaruvongvanich et al.'s meta-analysis did not find a significant correlation between serum vitamin D levels and NAFLD severity as indicated by NAFLD activity scores (NAS) and fibrosis stages^22^.

MetS is often viewed as a pivotal factor in NAFLD development. Several studies have correlated low serum vitamin D levels with MetS features^23,24^. Animal research further suggests that deficiency in hepatic vitamin D receptors, which regulate intra-hepatic lipid accumulation, or vitamin D insufficiency itself, impairs insulin secretion from pancreatic beta cells, highlighting vitamin D's beneficial role in diabetes and insulin resistance^25^. While the full NAFLD pathogenesis remains elusive, insulin resistance is considered a core mechanism in hepatic steatosis. The MAFLD acronym emphasizes metabolic factors in patients with hepatic steatosis. There is evidence suggesting that vitamin D insufficiency is directly associated with insulin resistance, especially in overweight and obese individuals, increasing MAFLD risk^26^. Current evidence supports vitamin D's potential role in MAFLD prevention by ameliorating aspects of MetS.

Yet, the extent to which vitamin D mitigates fatty liver development through MetS improvement alone is under scrutiny. With the evolution of NAFLD understanding from the "two-hit" to the "multiple-hits" hypothesis, FLD is seen as the result of several concurrent processes^27^. Vitamin D impacts these pathways non-classically, including hormonal, immune, and cellular differentiation mechanisms^28–30^. Our study delves into vitamin D's relationship with fatty liver in individuals with preexisting metabolic dysfunction. The observed benefits of vitamin D could stem from improvements in insulin resistance, elevation of adipokines like adiponectin, and reduction of hepatic inflammation. MAFLD in obesity is determined by changes in AT due to excessive caloric intake and weight gain^31^. Clinical trials and experiments indicate vitamin D treatment mitigates oxidative stress and local pro-inflammatory cytokine concentrations, such as tumor necrosis factor-ɑ (TNF-ɑ) and monocyte chemoattractant protein-1 (MCP-1)^32,33^. Vitamin D's systemic and tissue-specific anti-inflammatory properties are well documented^34^. In MAFLD rat models, active vitamin D treatment reduced liver inflammation and oxidative stress by inhibiting the p53-p21 pathway and associated cell senescence. Human studies have shown inverse correlations between VDR expression and the severity of steatosis and lobular inflammation^30^. Conversely, vitamin D insufficiency may exacerbate hepatic inflammation.

Another significant clinical observation is the more pronounced association between vitamin D insufficiency and MAFLD as vitamin D levels increase, suggesting a concentration-dependent preventive effect of vitamin D against MAFLD development. However, this inference warrants caution. Our findings also suggest that women with vitamin D insufficiency have higher odds for MAFLD incidence compared to men, supporting Bennouar's report of NAFLD association with vitamin D insufficiency in women irrespective of weight or metabolic profile^34^. Gender-specific differences in FLD development could be due to variations in circulating sex hormones and binding globulins^35^. Thus, the interplay of low vitamin D levels with high estradiol and low SHBG may contribute to the higher incidence of MAFLD in women. Additionally, gender differences emerged in the subgroup analyses, with consistent associations between vitamin D insufficiency and MAFLD in men across most subgroups, but not in women. Specifically, no link was found in women with hepatitis, diabetes, metabolic dysregulation, or those abstaining from alcohol. However, among heavy-drinking women, vitamin D had a more significant impact on MAFLD incidence than in men. Further research is needed to clarify these observations.

The conclusions of our study must be approached with circumspection due to several methodological limitations. Firstly, the cross-sectional design does not allow for definitive inferences regarding causality and may be prone to reverse causation between vitamin D insufficiency and MAFLD. Secondly, our study's reliance on the FLI as the sole predictor for hepatic steatosis was a pragmatic decision influenced by the large scale of our cohort and the standardized procedures of the health examinations undertaken. The FLI is an established indirect method for estimating liver fat content within large population groups. However, we recognize it is not a direct imaging method such as ultrasound, Fibroscan, or advanced techniques like magnetic resonance imaging derived proton density fat fraction (MRI-PDFF), which may offer more specificity. Although these diagnostic tools were not utilized, potentially affecting the precision of our fatty liver disease prevalence estimates, the FLI remains a vital tool for epidemiological insights, particularly in settings where direct imaging is impractical. Despite its limitations in detailing the complexities and differentiating the stages of NAFLD and NASH, the FLI is widely accepted for its non-invasive assessment capabilities.

Thirdly, while our study provides valuable insights into the relationship between vitamin D levels and the incidence of MAFLD, we must acknowledge that dietary patterns, which were not captured in our dataset, represent a potential confounding factor. Total calorie intake and diet composition are known to influence both vitamin D status and hepatic outcomes. The absence of this data limits our ability to fully account for dietary influences and necessitates cautious interpretation of the associations reported herein. Future investigations would benefit from incorporating detailed dietary assessments to delineate the independent effects of vitamin D from those of dietary habits on MAFLD outcomes. This approach would undoubtedly contribute to a more comprehensive understanding of the multifactorial nature of MAFLD and its potential nutritional determinants. The modest associations reported here between vitamin D insufficiency and MAFLD suggest that the role of vitamin D might be more complex and potentially moderated by a variety of factors, including but not limited to, nutritional intake, lifestyle, and genetic variability. This complexity is further compounded by the absence of direct imaging techniques in our study, a limitation which we have sought to address through careful interpretation and the consideration of additional confounders. Fourthly, being a single-center study, there's a possibility that the baseline characteristics of our subjects, who were selected based on routine health examinations, may not be representative of the broader population. This limits the extent to which our findings can be generalized. Lastly, the threshold we set for vitamin D insufficiency, at less than 20 ng/mL, could be contentious given the ongoing clinical debate and lack of a standardized definition for optimal vitamin D levels.

## Conclusion

Our comprehensive study has identified a significant relationship between vitamin D insufficiency and the incidence of MAFLD in individuals exhibiting metabolic dysfunction. Despite the inherent limitations of a cross-sectional and single-center design, these preliminary findings are noteworthy, suggesting an association that merits deeper investigation. Future studies, ideally featuring a more varied and longitudinal participant group, are essential to corroborate our findings and to determine the role of vitamin D more definitively in MAFLD pathogenesis.

## Methods

### Study design and population

**Figure 2:**
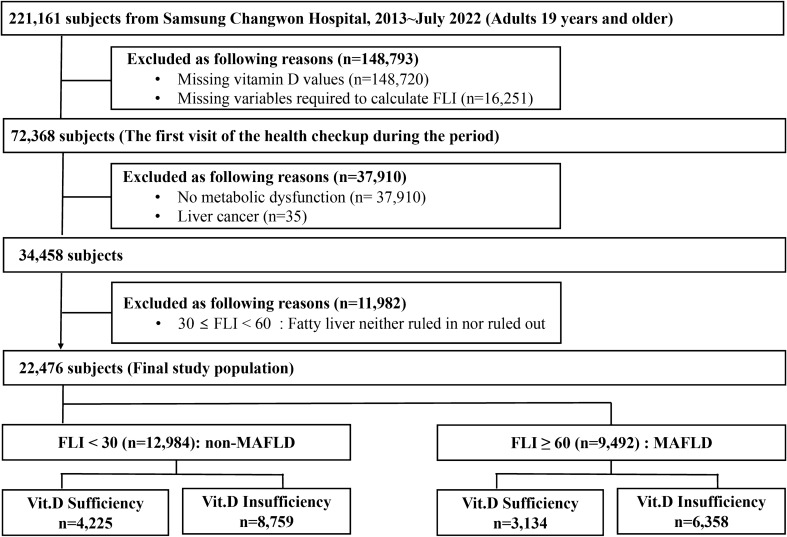
Flow diagram of the study population selection.

Our research included men and women aged 19 years and above who attended the Health Promotion Center at Samsung Changwon Hospital, Korea, for routine health examinations from January 2013 to July 2022, totaling 221,161 participants. For those with multiple health check-ups within this timeframe, the initial visit was designated as the baseline date. From this population, we excluded 148,793 individuals due to incomplete serum vitamin D measurements or missing FLI data, leaving 72,368 individuals suitable for fatty liver and vitamin D status assessment. A further exclusion of 37,910 subjects who did not fulfill the criteria for any of three metabolic conditions—diabetes mellitus, overweight/obesity, or metabolic dysregulation in normal weight—was performed to focus on subjects with metabolic dysfunction. Additionally, 35 individuals with a history of hepatic malignancy were also excluded from the study. To further refine our cohort, we excluded 11,982 subjects with an FLI between 30 to < 60, where the presence of fatty liver was ambiguous. Consequently, our final analytical cohort consisted of 22,476 participants who had evidence of metabolic dysfunction at baseline and available serum vitamin D status. Within this cohort, subjects with an FLI < 30 were categorized into the non-MAFLD group, while those with an FLI ≥ 60 were placed in the MAFLD group. Each group was then subdivided based on vitamin D levels into sufficiency and insufficiency subgroups for detailed analysis, as depicted in Fig. 2. The Institutional Review Board of Samsung Changwon Hospital (SCMC 2022-10-003) granted ethical approval for this study, which was conducted in line with the Declaration of Helsinki's guidelines. Given the study's retrospective nature, involving the analysis of pre-existing administrative and clinical data, the Institutional Review Board waived the requirement for informed consent from participants.

### Variables and measurements

For the anthropometric and physiological measurements, an InBody 770 body composition analyzer (Inbody Inc., Seoul, Korea) was employed, recording height, body weight, and WC. BMI was determined using the formula: weight in kilograms divided by the square of height in meters. Blood pressure was measured after a five-minute seated rest using an automated oscillometric sphygmomanometer, noting both systolic (SBP) and diastolic blood pressure (DBP). The self-reported health behavior questionnaire provided data on alcohol consumption, smoking habits, and physical activity. Alcohol consumption was categorized into never, moderate (less than 30 g/day for men and less than 20 g/day for women), or heavy (30 g/day or more for men and 20 g/day or more for women). Smoking status was classified as never, former, or current. Physical activity was divided into three tiers based on the International Physical Activity Questionnaires (IPAQ): inactive, moderately active, and health-enhancing physical activity (HEPA) active, with specific criteria for each category based on vigorous activity durations, moderate-intensity activities, walking, and MET-minutes per week^36^.

Laboratory measurements were conducted on blood samples collected after a minimum eight-hour overnight fast. The analysis included FPG, TC, LDL-C, TG, HDL-C, AST, ALT, and GGT. Vitamin D status was evaluated using a serum 25-OH-vitamin D3 Total Assay (Cobas e 602, Roche Diagnostics). HbA1c levels were measured by an HLC-723 G11 analyzer (Tosho, Inc). Additionally, serum insulin was assayed with commercial kits on an electrochemiluminescence device (Cobas e 602, Roche Diagnostics). Insulin resistance was calculated using the HOMA formula: HOMA-IR = fasting insulin (µIU/mL) * fasting glucose (mmol/L)/22.5^37^.

### Diagnostic criteria and definition of groups

Metabolic dysfunction within the study cohort was determined by the presence of one or more of the following criteria: (1) overweight or obesity, defined as a BMI of 23 kg/m^2^ or greater, following the Asia–Pacific guidelines; (2) T2DM; or (3) metabolic dysregulation characterized by at least two out of seven metabolic anomalies: WC ≥ 90 cm for men or ≥ 80 cm for women, prediabetes (HbA1c levels between 5.7% and 6.4%, FPG between 100 and 125 mg/dL, or 2-h post-load glucose levels between 140 and 199 mg/dL), hypertension or use of antihypertensive medications (blood pressure ≥ 130/85 mmHg), reduced high-density lipoprotein cholesterol (HDL-C < 40 mg/dL for men and < 50 mg/dL for women), elevated triglycerides (TG ≥ 150 mg/dL or on specific medication), insulin resistance as indicated by a HOMA-IR score ≥ 2.5, and elevated high-sensitivity C-reactive protein (hs-CRP > 2 mg/L).

FLI, a validated surrogate marker of hepatic steatosis, was utilized for predicting fatty liver disease (FLD). The FLI algorithm incorporates variables including BMI, TG, GGT levels, and WC, calculated as follows: FLI = ey / (1 + ey) * 100, y = 0.953 * loge (TG) + 0.139 * BMI + 0.718 * loge (GGT) + 0.053 * WC − 15.745. An FLI score below 30 excludes, scores from 30 to 60 are inconclusive for, and scores above 60 confirm the presence of fatty liver^38^.

Participants exhibiting metabolic dysfunction were dichotomized into the MAFLD and non-MAFLD groups based on their baseline FLI scores. Individuals with an FLI ≥ 60 were categorized under the MAFLD group, whereas those with an FLI < 30 were allocated to the non-MAFLD group. Subsequent classification into vitamin D sufficiency and insufficiency was based on serum levels, with the threshold set at 20 ng/mL, in alignment with the Food and Nutrition Board of the Institute of Medicine's definition of vitamin D insufficiency^39^.

### Statistical analysis

We utilized STATA software, version 14.0 (StataCorp LLC, College Station, Texas, USA), and R, version 3.4.4 (R Foundation for Statistical Computing, Vienna, Austria; URL: http://www.R-project.org/), to conduct our statistical analyses. To compare baseline characteristics between the groups with sufficient and insufficient vitamin D levels, we employed the independent t-test for continuous variables and the Chi-square test for categorical variables. Data are reported as mean ± standard deviation (SD) for continuous measures and as count and percentage (n, %) for categorical measures. The relationship between vitamin D insufficiency and the incidence of fatty liver disease in individuals with metabolic dysfunction was assessed using binary logistic regression models. To control for baseline confounders, we applied a series of models with incremental adjustments. Model 1 was adjusted for age, and Model 2 further included the year and quarter of the health checkup. Model 3 was expanded to adjust for alcohol consumption levels (never, moderate, or heavy). The final model, Model 4, included adjustments for physical activity levels (inactive, moderately active, or HEPA active) and smoking status (never, former, or current). Subgroup analyses were performed to investigate the impact of vitamin D status on the incidence of MAFLD within specific cohorts. Sensitivity analyses were also conducted using a lower FLI cut-off of 30, in recognition of studies suggesting a more appropriate threshold for the Korean population compared to Western cohorts. All statistical tests were two-sided, and a *P* value of less than 0.05 was considered to indicate statistical significance.

### Supplementary Information

Supplementary Information.

## Data Availability

The datasets generated and analyzed during this study are not publicly available due to restrictions pertaining to patient consent and institutional guidelines for data sharing. However, the datasets are available from the corresponding author upon reasonable request.
