# Supplementary material for: The contribution of vitamin D insufficiency to the onset of steatotic liver disease among individuals with metabolic dysfunction
Source: Sci Rep. 2024 Mar 20;14:6714. doi: 10.1038/s41598-024-57380-9 (PMC10954610; doi:10.1038/s41598-024-57380-9)
Supplement: Supplementary file 1 — Supplementary Information. [file 41598_2024_57380_MOESM1_ESM.pdf]

Supplementary Table 1. Incidence of MAFLD according to vitamin D status when FLI ≥30 is defined as MAFLD

	**aOR (95% CI)**	***P*-value**
**Men**		
Sufficiency	Reference	
Insufficiency	1.25 (1.17-1.34)	<0.001
**Women**		
Sufficiency	Reference	
Insufficiency	1.39 (1.18-1.64)	<0.001

Adjusted odds ratios and *P*-values were calculated using binary logistic regression. The model was adjusted for age, year and quarter of health checkup, alcohol consumption, physical activity, and smoking status. Abbreviation: aOR, adjusted odds ratio; FLI, fatty liver index; MAFLD, metabolic associated fatty liver disease
